# THE JOY AND CHALLENGES OF STORY CREATION WITH TIMESLIPS: STUDENT FACILITATOR PERCEPTIONS

**DOI:** 10.1093/geroni/igac059.407

**Published:** 2022-12-20

**Authors:** Emily Ihara, Emily Perez, Kendall Barrett, Megumi Inoue, Catherine Tompkins

**Affiliations:** George Mason University, Fairfax, Virginia, United States; George Mason University, Fairfax, Virginia, United States; George Mason University, Fairfax, Virginia, United States; George Mason University, Fairfax, Virginia, United States; George Mason University, Fairfax, Virginia, United States

## Abstract

Creative arts interventions for people living with dementia have been shown to improve mood, emotions, communication, and relationships for older people living with dementia and their care partners. Previous research demonstrates that TimeSlips, a creative storytelling intervention, provides a “failure-free” environment and an opportunity for individuals to use their imagination. Because it does not involve memory, people living with dementia are encouraged to contribute and interact, thus creating an environment that focuses on dignity and strengths rather than deficits. This case study explores the student facilitators’ experiences of running TimeSlips sessions in different levels of care. The two facilitators ran continuous sessions over six months – with an individual at home, group sessions in memory care, and group sessions in assisted living. The facilitators journaled about their experiences after each session and the same picture prompts were used across the different types of sessions. Each journal entry was coded by two independent researchers using grounded theory principles. Through the coding, it was clear that the facilitators needed to use different skills to engage participants in storytelling based on their level of care. Themes that emerged include joy of connection, playfulness, and engagement. In addition, specific skills are needed to accommodate some behaviors of people living with dementia (such as aggressive behavior toward others in the group) and how to distract from those behaviors to continue with the storytelling. The benefits and challenges of each modality and gaps that may need to be addressed for student facilitators will be discussed.

